# Sacituzumab Govitecan in Metastatic Triple-Negative Breast Cancer: A Systematic Review with Meta-Analysis of Single-Arm Efficacy and Integration of Randomized and Real-World Evidence

**DOI:** 10.3390/cancers18122005

**Published:** 2026-06-20

**Authors:** Marcelino Pérez-Bermejo, Marcelo Mazón-Albalate, María Teresa Murillo-Llorente, Javier Pérez-Murillo, María Ester Legidos-García, Francisco Tomás-Aguirre, Alma María Palau-Ferré, Miriam Martínez-Peris, Ignacio Ventura

**Affiliations:** 1 SONEV Research Group, Faculty of Medicine and Health Sciences, Catholic University of Valencia San Vicente Mártir, C/Quevedo No. 2, 46001 Valencia, Spain; mt.murillo@ucv.es (M.T.M.-L.); javier.perezmu@ucv.es (J.P.-M.); ester.legidos@ucv.es (M.E.L.-G.); paco.tomas@ucv.es (F.T.-A.); am.palau@ucv.es (A.M.P.-F.); miriam.martinez@ucv.es (M.M.-P.); 2 Faculty of Medicine and Health Sciences, Catholic University of Valencia San Vicente Mártir, C/Quevedo No. 2, 46001 Valencia, Spain; marcelo.mazon@mail.ucv.es (M.M.-A.); ignacio.ventura@ucv.es (I.V.); 3 Translational Research Center San Alberto Magno CITSAM, Catholic University of Valencia San Vicente Mártir, C/Quevedo No 2, 46001 Valencia, Spain

**Keywords:** triple-negative breast neoplasms, sacituzumab govitecan, antibody–drug conjugates, meta-analysis, progression-free survival, real-world evidence

## Abstract

Background/Objectives: Metastatic triple-negative breast cancer (mTNBC) carries a poor prognosis and limited treatment options. Sacituzumab govitecan (SG), an anti-Trop-2 antibody–drug conjugate, has shown activity in this setting, but a quantitative synthesis integrating randomized and real-world evidence—particularly in underrepresented subgroups—was lacking. We aimed to summarize the comparative benefit of SG, which derives from a single randomized trial, and to assess whether trial-level efficacy is consistent with the activity observed in routine practice. Methods: Following PRISMA 2020, we searched PubMed/MEDLINE and Web of Science (January 2017–April 2026) for trials and observational cohorts of SG monotherapy in mTNBC. Comparative effects were taken from randomized data; single-arm efficacy (objective response rate [ORR], clinical benefit rate [CBR], median progression-free [PFS], and overall survival [OS]) was pooled using random-effects models. Risk of bias was assessed with RoB 2 and ROBINS-I. The review was registered in the Open Science Framework. Results: Nine studies (1242 patients; 980 SG-treated) were included: one randomized trial (ASCENT), two single-arm trials, and six real-world cohorts. In ASCENT, SG improved PFS (hazard ratio [HR] 0.41, 95% CI 0.33–0.63) and OS (HR 0.51, 0.42–0.64). Pooled ORR was 31.1% (28.0–34.4), and CBR was 42.2% (37.7–46.8), with the median PFS being 4.8 months (4.4–5.3) and OS being 11.0 months (9.3–13.0); trial-derived and real-world estimates were concordant. The benefit persisted in older patients (HR 0.25) and Black women (HR 0.44) but not in those with brain metastases (HR 0.68, 0.38–1.23). Conclusions: SG shows clinically meaningful activity in mTNBC that is broadly consistent between controlled trials and routine practice. Comparative superiority over chemotherapy rests on a single randomized trial (ASCENT); the pooled single-arm estimates describe activity and its consistency rather than a comparative effect.

## Simple Summary

Triple-negative breast cancer is an aggressive form of breast cancer that often spreads to other parts of the body, where it becomes very difficult to treat, and survival is usually short. For many years, standard chemotherapy was almost the only option, and its benefit rarely lasted long. A newer medicine called sacituzumab govitecan was designed to carry a cancer-killing agent directly to tumor cells while causing less harm to healthy tissue. We gathered and combined the results of every available clinical trial and of patients treated in everyday hospital practice to measure how well this medicine shrinks the cancer and helps people live longer, and to examine whether the results seen in trials also hold in routine care. We also examined whether it helps groups often underrepresented in trials, such as older women, women of African ancestry, and patients whose cancer has reached the brain. Our findings can inform treatment decisions and guide future research.

## 1. Introduction

Triple-negative breast cancer (TNBC) accounts for approximately 10–15% of all breast carcinomas and is defined by the absence of estrogen and progesterone receptors together with the lack of HER2 overexpression—a phenotype that precludes both endocrine and HER2-directed therapy and confers the poorest prognosis among breast cancer subtypes [1,2,3]. The disease is disproportionately frequent in women of African ancestry, tends to arise at a younger age, and is enriched for BRCA1 and TP53 alterations and high proliferative activity, as reflected by an elevated Ki-67 index [4,5,6]. Once metastatic, mTNBC follows an aggressive clinical course, and for decades, sequential single-agent cytotoxic chemotherapy remained the only systemic option, yielding low response rates, short-lived disease control, and substantial toxicity [7]. Over the past decade, biomarker-defined options have emerged for selected patients—immune checkpoint inhibitors combined with chemotherapy for programmed death-ligand 1 (PD-L1)–positive disease [8,9,10] and poly(ADP-ribose) polymerase (PARP) inhibitors for germline BRCA-mutated tumors [11,12], yet most patients are ineligible for these therapies or are unlikely to see progress and continue to depend on cytotoxic chemotherapy, leaving a substantial unmet need [13].

**Figure 1:**
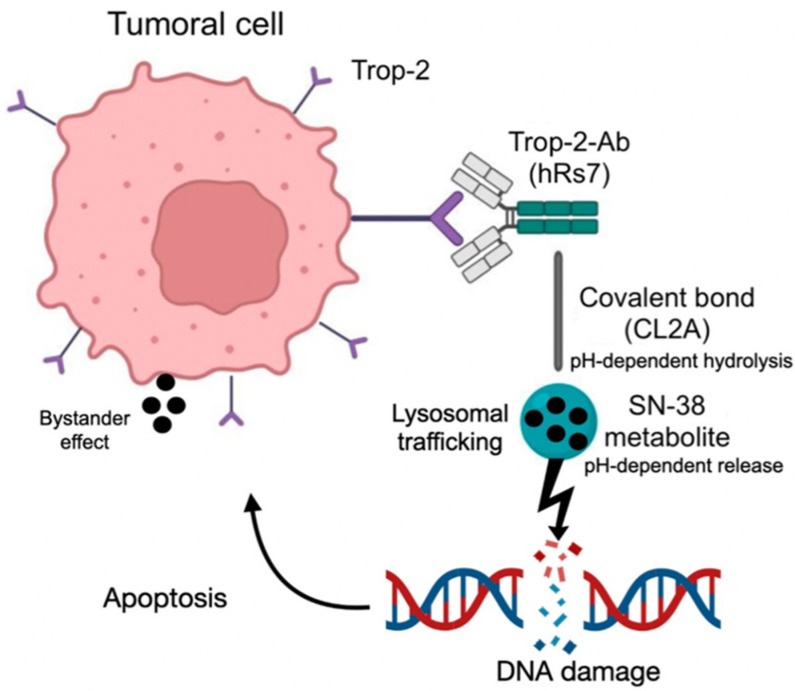
Mechanism of action of sacituzumab govitecan. Sacituzumab govitecan is an antibody–drug conjugate in which a humanized anti-Trop-2 monoclonal antibody (hRS7) is conjugated to SN-38, the active metabolite of irinotecan, via a hydrolyzable, pH-sensitive CL2A linker at a high drug-to-antibody ratio (~7.6). Following selective binding to Trop-2 receptors overexpressed on triple-negative breast cancer cells, the conjugate undergoes receptor-mediated internalization and lysosomal trafficking. pH-dependent hydrolysis of the linker releases SN-38 both intracellularly and in the acidic tumor microenvironment. As a potent topoisomerase I inhibitor, SN-38 induces DNA damage leading to apoptotic cell death. Because SN-38 is membrane-permeable, it can also diffuse into adjacent cells irrespective of their Trop-2 expression, mediating a bystander effect that enhances antitumor activity in tumors with heterogeneous Trop-2 expression. Compared with conventional chemotherapy, this targeted design is intended to concentrate cytotoxic activity within the tumor while reducing, but not eliminating, systemic exposure. Abbreviations: ADC, antibody–drug conjugate; Trop-2, trophoblast cell-surface antigen 2; SN-38, 7-ethyl-10-hydroxycamptothecin.

Antibody–drug conjugates (ADCs) have reshaped this therapeutic landscape by uncoupling cytotoxic potency from systemic exposure [14,15,16]. Sacituzumab govitecan (SG) is a first-in-class ADC in which a humanized anti-Trop-2 monoclonal antibody (hRS7) is linked through a hydrolyzable CL2A linker to SN-38, which is the active metabolite of irinotecan [14,15,16]. Trophoblast cell-surface antigen 2 (Trop-2) is overexpressed in the majority of TNBC tumors, where it promotes proliferation, invasion, and an epithelial–mesenchymal transition, making it a particularly attractive target [17,18,19]. Antibody binding triggers receptor-mediated internalization and intracellular release of SN-38, a potent topoisomerase I inhibitor that induces DNA damage and apoptosis [20,21,22]. A bystander effect—whereby membrane-permeable SN-38 diffuses into neighboring cells irrespective of their Trop-2 expression—further amplifies antitumor activity across heterogeneous tumor microenvironments, while the targeted design limits systemic cytotoxic exposure (Figure 1) [23,24].

This rationale has been borne out clinically. The phase I/II IMMU-132-01 basket trial first demonstrated durable responses in heavily pretreated mTNBC, and the randomized phase III ASCENT subsequently established the superiority of SG over single-agent chemotherapy of the physician’s choice in progression-free survival (PFS), objective response rate (ORR), and overall survival (OS), leading to its regulatory approval and guideline endorsement as a preferred option in the second-line and later setting [25,26,27]. Nonetheless, important questions remain. Because pivotal trials enroll selected populations, the magnitude of benefit in clinically relevant subgroups—patients with brain metastases, older adults (≥65 years), and women of African ancestry, who bear a disproportionate share of TNBC incidence and mortality—is incompletely characterized, as is the consistency of trial-level efficacy in routine clinical practice. Moreover, prior quantitative syntheses have largely been single-arm or have not integrated the rapidly expanding body of real-world evidence, leaving the comparative benefit of SG across these dimensions unresolved.

To address these gaps, we conducted a systematic review and meta-analysis integrating randomized controlled trials and real-world observational studies. Because only one randomized trial provides a concurrent comparator, our primary aim was to pool the single-arm activity of SG monotherapy (ORR, CBR, and median PFS and OS) across controlled trials and routine practice and to assess its consistency between these settings; the comparative benefit of SG versus chemotherapy was taken from that single randomized trial (ASCENT) and was not pooled. We placed prespecified emphasis on clinically relevant subgroups.

## 2. Materials and Methods

### 2.1. Protocol, Registration, and Reporting

This systematic review with meta-analysis was designed, conducted, and reported in accordance with the Preferred Reporting Items for Systematic Reviews and Meta-Analyses (PRISMA) 2020 statement [28]. The review was registered in the Open Science Framework (OSF) under DOI 10.17605/OSF.IO/EN5TK. The completed checklist is provided in Supplementary Table S1.

### 2.2. Research Question and Eligibility Criteria

The review question was framed using the PICOS structure. Eligible studies enrolled adult patients (≥18 years) with histologically confirmed metastatic triple-negative breast cancer (mTNBC) [population] treated with sacituzumab govitecan (SG) as monotherapy [intervention]. For comparative synthesis, the comparator was the standard single-agent chemotherapy or treatment of the physician’s choice (TPC) [comparison]; single-arm studies without a concurrent comparator were eligible for descriptive single-arm synthesis only. The outcomes of interest [outcomes] were progression-free survival (PFS) and overall survival (OS) as co-primary outcomes and objective response rate (ORR) and clinical benefit rate (CBR) as secondary outcomes. Eligible designs [study designs] were randomized controlled trials (RCTs) and prospective or retrospective observational cohort studies, including real-world cohorts.

Studies were included when they (1) evaluated SG monotherapy in mTNBC; (2) reported at least one outcome of interest as an effect estimate with its 95% confidence interval (CI)—a hazard ratio (HR) for time-to-event outcomes, or an odds ratio (OR), risk ratio, or extractable event counts for binary outcomes—or provided Kaplan–Meier curves permitting the reconstruction of these estimates; and (3) were published between January 2017, the year of the first clinical report of SG in mTNBC, and 30 April 2026. We excluded studies of SG in combination regimens, populations that were not confined to mTNBC (unless mTNBC-specific data were extractable), case reports, narrative reviews, editorials, and conference abstracts lacking extractable quantitative data. Inclusion was restricted to reports written in English, Spanish, or German.

### 2.3. Information Sources and Search Strategy

We systematically searched PubMed/MEDLINE and the Web of Science Core Collection from January 2017 to 30 April 2026. These two databases were selected for their broad, complementary coverage of the oncology literature and their substantial overlap with Embase and the Cochrane Central Register of Controlled Trials (CENTRAL) for breast-cancer trials, supplemented by hand searching and congress screening to mitigate the risk of missing eligible reports; this pragmatic choice, together with the restriction to English, Spanish, and German, is explicitly acknowledged as a limitation in the Discussion. The search combined controlled vocabulary (Medical Subject Headings) and free-text terms for the population and intervention, for example: (“Triple Negative Breast Neoplasms” OR “triple-negative breast cancer”) AND (“Sacituzumab Govitecan” OR “Trodelvy” OR “IMMU-132” OR “anti-Trop-2 antibody–drug conjugate”). The complete, database-specific search strategies, including all filters and the exact dates of execution, are provided in Supplementary Table S2. We additionally hand-searched the reference lists of all included studies and relevant systematic reviews, and screened the proceedings of major oncology congresses (ASCO, ESMO, and SABCS) to identify eligible full reports.

### 2.4. Study Selection

All records were imported into reference-management software and de-duplicated, first automatically and then manually. Two reviewers independently screened titles and abstracts and subsequently assessed the full text of potentially eligible records against the predefined criteria. Disagreements were resolved by discussion or, when consensus could not be reached, by a third senior reviewer. Inter-reviewer agreement at the full-text stage was quantified using Cohen’s κ. The selection process is summarized in the PRISMA 2020 flow diagram.

### 2.5. Data Extraction

Two reviewers independently extracted data using a standardized, pre-piloted form, with discrepancies reconciled by consensus. Extracted variables included the first author, year of publication, country, study design, sample size, line of therapy, relevant demographic and clinical characteristics (age, race or ethnicity, presence of brain metastases, and Trop-2 status when reported), treatment details, and outcome data. For time-to-event outcomes, reported HRs and 95% CIs were extracted directly; when these were not available, they were estimated from published Kaplan–Meier curves and numbers at risk using the methods of Tierney et al. [29] and the Guyot algorithm [30] for reconstruction of individual time-to-event data. For binary outcomes, event counts were extracted to derive ORs and risk ratios with 95% CIs. Corresponding authors were contacted in the event of missing or unclear data.

### 2.6. Outcome Definitions

PFS was defined as the time from treatment initiation to documented disease progression or death from any cause, and OS was defined as the time from treatment initiation to death from any cause. ORR was defined as the proportion of patients achieving a complete or partial response according to Response Evaluation Criteria in Solid Tumors (RECIST) version 1.1, and CBR as the proportion achieving a complete response, partial response, or stable disease lasting at least 6 months.

### 2.7. Risk-of-Bias Assessment

Two reviewers independently appraised methodological quality using design-appropriate instruments: the Cochrane Risk of Bias 2 (RoB 2) tool for RCTs [31], covering five domains (the randomization process, deviations from intended interventions, missing outcome data, measurement of the outcome, and selection of the reported result), and the Risk Of Bias In Non-randomized Studies of Interventions (ROBINS-I) tool for observational studies [32], covering seven domains (confounding, selection of participants, classification of interventions, deviations from intended interventions, missing data, measurement of outcomes, and selection of the reported result). Each study was rated as being at low, moderate (some concerns), or high (serious) risk of bias. These judgments informed the sensitivity analyses and the GRADE assessment of certainty. The full domain-level appraisals are reported in Supplementary Table S3 (RoB 2, ASCENT) and Supplementary Table S4 (ROBINS-I, non-randomized studies).

### 2.8. Data Synthesis and Statistical Analysis

Quantitative synthesis was performed separately for each outcome. Because clinical and methodological diversity across pivotal trials and real-world cohorts was anticipated, random-effects models were used throughout. Between-study variance (τ^2^) was estimated using the DerSimonian-Laird method, with restricted maximum likelihood (REML) applied as a sensitivity estimator. Given the small number of studies available for several outcomes, the 95% CIs of the pooled estimates were additionally derived using the Hartung–Knapp–Sidik–Jonkman adjustment [33], which yields more conservative intervals than the conventional normal approximation when few studies are pooled. All effects were synthesized on the logarithmic scale and exponentiated for presentation; HRs were pooled by generic inverse-variance weighting and ORs by the inverse-variance method. Median PFS and median OS were pooled on the natural-logarithmic scale using inverse-variance weights derived from the reported 95% confidence intervals, an approach that assumes approximately log-normally distributed survival times and that necessarily ignores between-study differences in follow-up duration, censoring pattern, line of therapy, and imaging schedules. We did not have access to individual patient data and therefore could not reconstruct or adjust time-to-event curves; the pooled medians should accordingly be read as a descriptive summary of central tendency rather than as an adjusted survival estimate, and we report 95% prediction intervals to convey the dispersion expected across settings. To examine the influence of study design on these summaries, median PFS and OS were additionally stratified into clinical trials and real-world cohorts, with a test for differences between subgroups, and the forest plots for the time-to-event outcomes are presented in this stratified for (Supplementary Table S5). The robustness of the pooled medians was confirmed in two prespecified sensitivity analyses: re-estimating the between-study variance by restricted maximum likelihood (REML) instead of DerSimonian-Laird changed the pooled medians negligibly (PFS 4.8 vs. 4.8 months; OS 10.9 vs. 11.0 months), and the leave-one-out analysis yielded narrow ranges (PFS 4.7–5.0 months; OS 10.2–11.8 months), with no single cohort altering the conclusions.

Comparative pooling (SG versus chemotherapy) was restricted to studies providing a concurrent comparator, namely RCTs and comparative cohorts. Single-arm studies were analyzed separately by pooling proportions (for example, ORR) with a generalized linear mixed model on the logit scale, and were not combined with comparative effect estimates. This separation prevents non-comparative response proportions from inflating the pooled comparative effects.

Statistical heterogeneity was quantified using Cochran’s Q test (with significance set at *p* < 0.10), the I^2^ statistic (interpreted as low, moderate, and high at approximately 25%, 50%, and 75%, respectively), and τ^2^. For the co-primary outcomes, 95% prediction intervals are reported to convey the range of treatment effects expected in future settings, which is particularly informative when real-world and trial populations are combined.

Prespecified subgroup analyses examined effect modification by the presence of brain metastases, age (≥65 versus <65 years), African ancestry or Black race, line of therapy, and study design (RCT versus real-world). Differences between subgroups were tested using the Q test for subgroup interaction, and random-effects meta-regression was planned for moderators when at least 10 studies were available. The robustness of the pooled estimates was assessed through leave-one-out sensitivity analyses and by restricting analyses to studies at low risk of bias.

Small-study effects and potential publication bias were assessed by visual inspection of funnel plots and, when at least 10 studies contributed to an outcome, by Egger’s regression test; with fewer studies, formal testing was not undertaken because of insufficient statistical power. All analyses were conducted in R version 4.3 (using the meta and metafor packages) and cross-checked in Meta-Essentials version 1.7. All tests were two-sided, and statistical significance was set at *p* < 0.05.

### 2.9. Certainty of Evidence

The certainty of the pooled evidence for each outcome was rated as high, moderate, low, or very low using the Grading of Recommendations, Assessment, Development and Evaluations (GRADE) framework [34], considering risk of bias, inconsistency, indirectness, imprecision, and publication bias. Results are summarized in a Summary of Findings table (Supplementary Table S6).

## 3. Results

### 3.1. Study Selection and Characteristics

**Figure 2:**
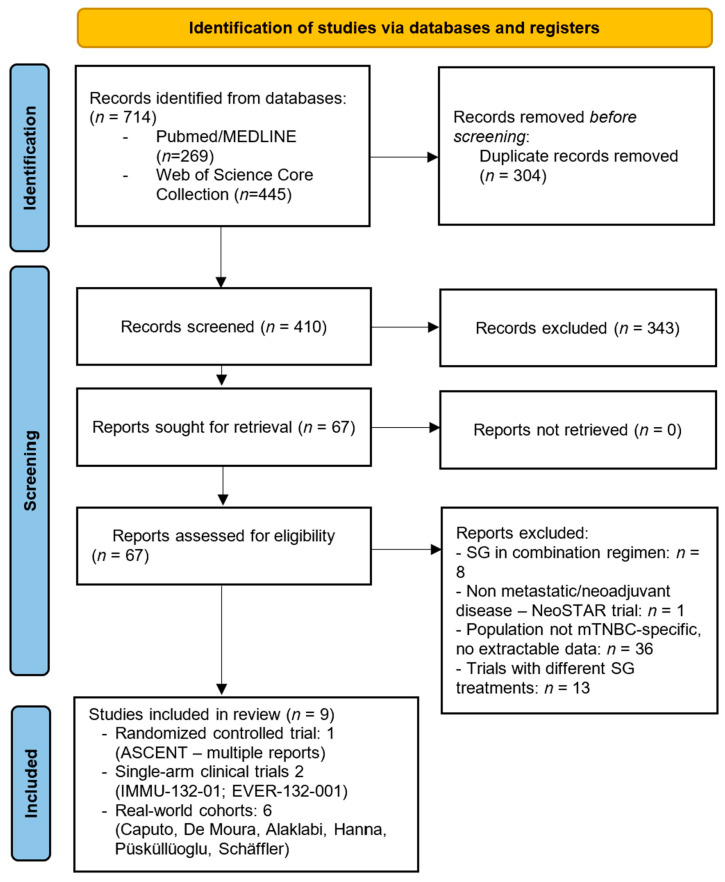
PRISMA 2020 flow diagram for study identification, screening, and inclusion.

The systematic search and citation screening yielded 714 records; after removal of duplicates and title/abstract screening, the full-text reports of all potentially eligible records were assessed for eligibility. Nine unique studies met the inclusion criteria and were included in the quantitative synthesis (Figure 2).

One report of the NeoSTAR trial was excluded because it evaluated neoadjuvant SG in non-metastatic, early-stage disease, outside the target mTNBC population [35]. Several publications reported overlapping data from the ASCENT—the pivotal reports [26,27] together with prespecified subgroup, biomarker, and secondary analyses [36,37,38,39,40,41,42]—and were treated as a single study to preserve statistical independence (Supplementary Table S7); the final database-lock report [27] provided the primary comparative estimates, and the consolidated subgroup report [36] the subgroup effects. Likewise, the phase I/II IMMU-132-01 trial, reported in an initial [43] and an expanded [25] analysis and in the regulatory approval summary [44], was consolidated into a single study.

The nine included studies comprised one randomized controlled trial (the phase III ASCENT [26,27]), two single-arm clinical trials (the phase I/II IMMU-132-01 basket trial [25] and the phase IIb EVER-132-001 trial in Chinese patients [45]), and six retrospective real-world cohorts from Italy, France, the United States, the United Kingdom, Poland, and Germany [46,47,48,49,50,51]. Together, they contributed 980 SG-treated patients and, within ASCENT, 262 patients receiving treatment of the physician’s choice (TPC). Their characteristics are summarized in Table 1. Only ASCENT provided a concurrent randomized comparator; accordingly, comparative (SG vs. chemotherapy) effects were derived from that trial alone, whereas single-arm efficacy was pooled across all SG-treated cohorts.

### 3.2. Comparative Efficacy: Randomized Evidence (ASCENT)

In the only randomized comparison, SG significantly prolonged PFS relative to TPC (median 4.8 vs. 1.7 months; hazard ratio [HR] 0.41, 95% CI 0.33–0.63) and OS (median 11.8 vs. 6.9 months; HR 0.51, 95% CI 0.42–0.64) [27]. Response outcomes also favored SG: the ORR was 31.1% with SG versus 4.2% with TPC (odds ratio [OR] 10.3, 95% CI 5.3–19.9), and the CBR was 40.4% versus 8.0% (OR 7.8, 95% CI 4.7–13.0). As comparative evidence derived from a single trial, these effects were not pooled.

### 3.3. Pooled Single-Arm Efficacy Across Trials and Real-World Cohorts

**Figure 3:**
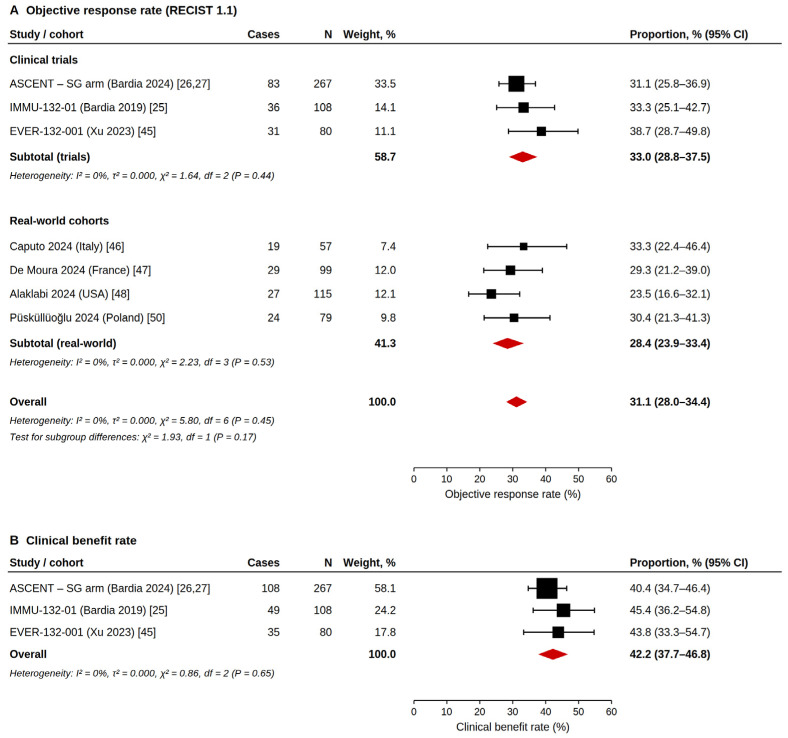
Random-effects meta-analysis of single-arm efficacy: (**A**) objective response rate and (**B**) clinical benefit rate. Black squares represent individual cohorts (size proportional to weight); red diamonds represent pooled estimates (DerSimonian-Laird, logit transformation). Panel A is stratified into clinical trials and real-world cohorts. Panel **A** is stratified into clinical trials and real-world cohorts. The cohorts included in (**A**) were ASCENT [26,27], IMMU-132-01 [25], EVER-132-001 [45], Caputo et al. [46], De Moura et al. [47], Alaklabi et al. [48], and Püsküllüoğlu et al. [50]; the cohorts included in (**B**) were ASCENT [26,27], IMMU-132-01 [25], and EVER-132-001 [45].

**Figure 4:**
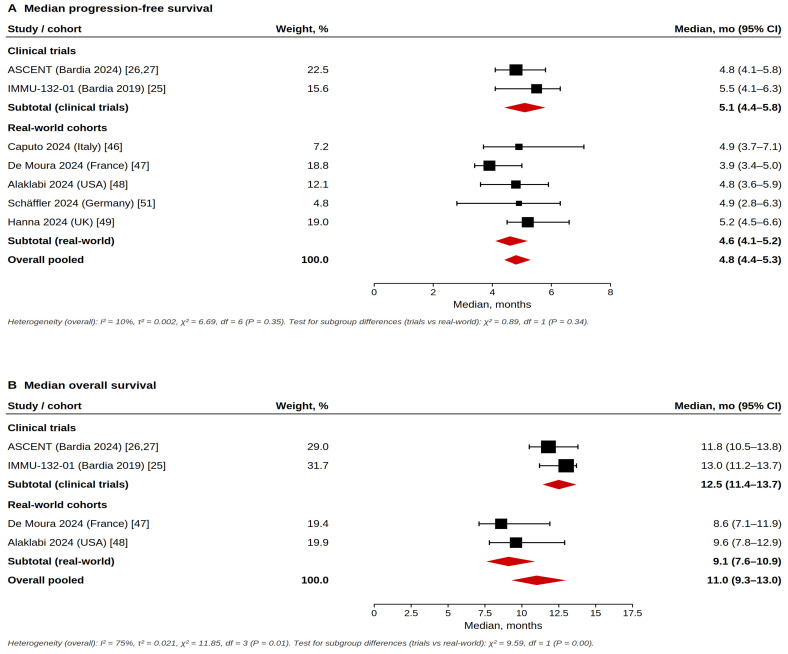
Random-effects meta-analysis of survival outcomes: (**A**) median progression-free survival and (**B**) median overall survival. Black squares represent individual cohorts (size proportional to weight); red diamonds represent the pooled estimates (DerSimonian–Laird random-effects model); horizontal lines denote the 95% confidence intervals. Both panels are stratified into clinical trials and real-world cohorts. The cohorts included in (**A**) were ASCENT [26,27], IMMU-132-01 [25], Caputo et al. [46], De Moura et al. [47], Alaklabi et al. [48], Schäffler et al. [51], and Hanna et al. [49]; the cohorts included in (**B**) were ASCENT [26,27], IMMU-132-01 [25], De Moura et al. [47], and Alaklabi et al. [48].

Across the seven cohorts reporting tumor response, the pooled ORR was 31.1% (95% CI 28.0–34.4), with no detectable heterogeneity (I^2^ = 0%; τ^2^ = 0; Figure 3A). The pooled CBR, available from three cohorts, was 42.2% (95% CI 37.7–46.8; I^2^ = 0%; Figure 3B). The pooled median PFS across seven cohorts was 4.8 months (95% CI 4.4–5.3; I^2^ = 10%; Figure 4A), and the pooled median OS across four cohorts was 11.0 months (95% CI 9.3–13.0), with substantial heterogeneity (I^2^ = 75%; Figure 4B) attributable to differences in prior treatment burden and follow-up between trial and real-world populations.

### 3.4. Reproducibility in Routine Practice (Trial vs. Real-World)

Efficacy was consistent between controlled trials and routine practice. The pooled ORR was 33.0% (95% CI 28.8–37.5) in clinical trials and 28.4% (95% CI 23.9–33.4) in real-world cohorts, with overlapping confidence intervals and no significant subgroup difference (Figure 3A). Median PFS was similarly concordant (trial point estimates 4.8–5.5 vs. real-world 3.9–5.2 months). Median OS tended to be lower in some real-world cohorts (8.6–9.6 months) than in trials (11.8–13.0 months), consistent with the more heavily pretreated, less selected real-world populations and explaining the OS heterogeneity. The design-stratified pooled medians and the test for subgroup differences are reported in Supplementary Table S5 (see also Figure 4).

### 3.5. Prespecified Subgroups (ASCENT)

**Figure 5:**
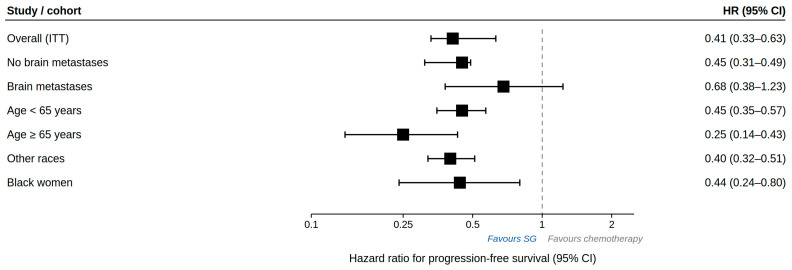
Prespecified subgroup analyses of progression-free survival within ASCENT (descriptive, not pooled). SG vs. treatment of physician’s choice; hazard ratios < 1 favor SG. Black squares denote the hazard-ratio point estimate for each subgroup and horizontal lines denote the corresponding 95% confidence interval; the vertical grey dashed line marks the line of no effect (HR = 1, i.e., no difference between SG and TPC). These are the trial’s prespecified subgroups as reported by Hurvitz et al. [36]; the estimates derive from a single randomized trial, are based on small numbers, and are shown to contextualize the effect in vulnerable subgroups, not as a meta-analytic synthesis. The brain-metastasis estimate did not reach statistical significance.

The estimates in this section are the prespecified subgroup analyses of the ASCENT reported by Hurvitz et al. [36]; they are presented descriptively to contextualize the effect of SG in clinically vulnerable subgroups and are not a meta-analytic synthesis of multiple studies. The PFS benefit of SG was preserved across clinically relevant subgroups of ASCENT (Figure 5). The effect was largest in patients aged ≥65 years (HR 0.25, 95% CI 0.14–0.43) and consistent in younger patients (HR 0.45, 95% CI 0.35–0.57). Black women derived a significant benefit (HR 0.44, 95% CI 0.24–0.80), comparable to other racial groups (HR 0.40, 95% CI 0.32–0.51). In patients with baseline brain metastases, the point estimate favored SG but did not reach significance (HR 0.68, 95% CI 0.38–1.23), in contrast to the clear benefit in patients without brain metastases (HR 0.45, 95% CI 0.31–0.49). Overall survival subgroup estimates followed the same direction (e.g., age ≥65: HR 0.47, 95% CI 0.29–0.75), although several did not reach significance owing to small subgroup sizes [36,37,38,42]. These subgroup estimates derive from a single randomized trial, are based on small numbers, and are subject to multiplicity; they are therefore hypothesis-generating rather than confirmatory. In particular, the brain-metastasis estimate did not reach statistical significance and should not be interpreted as evidence of benefit in this subgroup.

### 3.6. Safety Profile Across Included Studies

Although safety was not a prespecified endpoint of this efficacy-focused synthesis, the tolerability data reported by the nine included studies were extracted descriptively (Table 2). The dominant grade ≥3 toxicity was neutropenia, reported in approximately 29–62% of patients—highest in the Chinese EVER-132–001 cohort, consistent with a higher prevalence of UGT1A1 variants that impair SN-38 detoxification—whereas grade ≥3 diarrhea was generally below 15% and high-grade peripheral neuropathy was negligible (no grade 3–4 events in ASCENT). Febrile neutropenia occurred in roughly 6–9% of patients in the trials. Dose reductions for toxicity ranged from 17% to 54%, but treatment discontinuation attributable to adverse events was uncommon (approximately 1–13%), and treatment-related deaths were rare or absent across the included reports. The profile was therefore broadly consistent between controlled trials and routine practice and aligns with the established, manageable safety signature of SG, dominated by myelosuppression and gastrointestinal toxicity.

### 3.7. Heterogeneity, Sensitivity, and Risk of Bias

Heterogeneity was negligible for the response endpoints and PFS (I^2^ ≤ 10%) and high for OS (I^2^ = 75%). Leave-one-out analyses confirmed the robustness of the pooled estimates: omission of any single cohort changed the pooled ORR by ≤1.1 percentage points (range 30.2–32.2%) and did not materially affect the pooled CBR or median PFS (Supplementary Table S8). Because fewer than ten cohorts contributed to any single outcome, funnel-plot asymmetry and Egger’s test were not formally evaluated, and small-study effects cannot be excluded. On risk-of-bias appraisal, ASCENT was rated at low overall risk, with “some concerns” limited to its open-label design (key time-to-event outcomes were supported by blinded independent central review). The single-arm trials and real-world cohorts were rated at moderate-to-serious risk on ROBINS-I, driven mainly by the absence of a concurrent comparator and the residual confounding inherent to retrospective designs; these judgments informed the GRADE certainty ratings.

## 4. Discussion

This systematic review synthesized the available evidence on sacituzumab govitecan (SG) in metastatic triple-negative breast cancer (mTNBC) from nine unique studies: one randomized controlled trial (ASCENT), two single-arm clinical trials (IMMU-132-01 and EVER-132-001), and six retrospective real-world cohorts. Comparative superiority over chemotherapy is established by the single randomized trial: in ASCENT, SG prolonged progression-free survival (PFS; median 4.8 vs. 1.7 months; hazard ratio [HR] 0.41, 95% CI 0.33–0.63) and overall survival (OS; median 11.8 vs. 6.9 months; HR 0.51, 95% CI 0.42–0.64), with a higher objective response (31% vs. 4%; odds ratio [OR] 10.3, 95% CI 5.3–19.9) and clinical benefit (40% vs. 8%; OR 7.8, 95% CI 4.7–13.0) rates [26,27]. The principal contribution of our quantitative synthesis is not a re-estimation of this comparative effect—which rests on a single trial and cannot be meta-analytically pooled—but a pooled, single-arm estimate of SG activity across controlled trials and routine practice: an objective response rate (ORR) of 31.1% (95% CI 28.0–34.4), a clinical benefit rate (CBR) of 42.2% (95% CI 37.7–46.8), a pooled median PFS of 4.8 months (95% CI 4.4–5.3), and a pooled median OS of 11.0 months (95% CI 9.3–13.0). The low heterogeneity observed for response and PFS (I^2^ ≤ 10%) indicates that the activity seen in the pivotal trial is broadly consistent with that observed in unselected, real-world populations.

This broad consistency is the most clinically relevant message of the analysis. Randomized trials establish internal validity, but their restrictive eligibility criteria often exclude patients with comorbidities, poorer performance status, or extensive prior therapy. The close concordance between trial-derived and real-world response (pooled ORR 33.0% vs. 28.4%, with overlapping confidence intervals and no significant subgroup difference) and the comparable median PFS support the external validity of SG’s benefit. Median OS, by contrast, was somewhat shorter and considerably more heterogeneous across real-world cohorts (I^2^ = 75%; pooled 11.0 months) than in the trials, a difference plausibly explained by the more heavily pretreated, less selected real-world populations and by varying follow-up. This nuance—that response and PFS translate faithfully to practice, whereas survival is more context-dependent—is precisely the kind of information that trial data alone cannot provide, and that is directly useful to health technology assessment and guideline bodies.

The shorter and more variable real-world overall survival warrants a cautious, bias-aware interpretation. The six observational cohorts were retrospective and non-comparative, and they differed from the trial populations in ways that plausibly shorten survival independently of drug efficacy—a greater burden of prior therapy, poorer performance status, a higher prevalence of brain metastases, and unequal access to subsequent active lines. Because no patient-level adjustment was possible, these factors act as uncontrolled confounders, and the lower real-world OS is at least as likely to reflect selection and confounding by indication as any true attenuation of efficacy. We accordingly rated the real-world evidence at moderate-to-serious risk of bias on ROBINS-I and downgraded its GRADE certainty, and we read the trial-versus-real-world OS difference (Figure 4B) as descriptive rather than as evidence that SG is less effective in routine care.

The randomized subgroup analyses of ASCENT suggest that the PFS benefit is broadly maintained across clinically vulnerable populations, including older patients (age ≥65 years: HR 0.25, 95% CI 0.14–0.43), Black women (HR 0.44, 95% CI 0.24–0.80), and patients without brain metastases (HR 0.45, 95% CI 0.31–0.49) [36,38,42]. An important correction to earlier interpretations concerns brain metastases: although the point estimate numerically favored SG (median PFS 2.8 vs. 1.6 months), the effect did not reach statistical significance (HR 0.68, 95% CI 0.38–1.23), and the corresponding OS estimate showed no clear advantage (HR 0.96, 95% CI 0.55–1.68) [37]. These subgroup findings derive from a single trial, are based on small numbers, and are subject to multiplicity; they are therefore hypothesis-generating rather than confirmatory. Nonetheless, the consistent direction of effect in historically under-served groups is encouraging and supports dedicated evaluation, particularly of central nervous system-active strategies for patients with brain involvement.

These results must be read against a therapeutic landscape that has changed substantially since SG was first established in pretreated mTNBC. In 2025, two phase III trials extended SG into the first-line setting: ASCENT-03 compared SG with chemotherapy in patients ineligible for PD-(L)1 inhibitors and showed a significant PFS gain (median 9.7 vs. 6.9 months; HR 0.62, 95% CI 0.50–0.78) [52], while ASCENT-04/KEYNOTE-D19 combined SG with pembrolizumab in PD-L1-positive disease and improved PFS over chemotherapy plus pembrolizumab (median 11.2 vs. 7.8 months; HR 0.65, 95% CI 0.51–0.84) [53]. In parallel, a second Trop-2-directed antibody–drug conjugate, datopotamab deruxtecan, demonstrated first-line superiority over chemotherapy in immunotherapy-ineligible mTNBC in TROPION-Breast02 (PFS median 10.8 vs. 5.6 months; HR 0.57; with an OS benefit) and has since gained first-line approval [54]. Our synthesis, therefore, consolidates the second-line-and-beyond experience with SG at the very moment that Trop-2 ADCs are moving into earlier lines. The real-world consistency documented here offers a useful benchmark against which the durability and generalizability of these first-line gains can be assessed, and it sharpens unresolved questions about the optimal sequencing of Trop-2 ADCs and the management of post-ADC resistance. ASCENT-03 is particularly relevant for patients who are not candidates for immune checkpoint inhibitors—a large subgroup with few targeted options—and who position SG as a potential first-line standard in that setting; this may become one of its most clinically important indications. As SG and other anti-Trop-2 ADCs (notably datopotamab deruxtecan) move into the first line, the second-line-and-beyond activity summarized here will increasingly inform sequencing decisions rather than initial choice, and the key open questions become whether anti-Trop-2 ADCs retain activity after prior ADC exposure, how SG and datopotamab deruxtecan should be ordered, and which biomarkers beyond Trop-2 expression predict benefit. Our pooled estimates thus provide a real-world reference point for interpreting these first-line results and for designing the sequencing trials that the current evidence cannot yet answer.

Although safety was not a prespecified endpoint of this efficacy-focused synthesis, the tolerability profile reported across the included studies is relevant to interpretation. As summarized descriptively in Section 3.6 and Table 2, the dominant grade ≥3 toxicity across the nine studies was neutropenia (approximately 29–62%), grade ≥3 diarrhea was generally below 15%, febrile neutropenia affected roughly 6–9% of trial patients, dose reductions ranged from 17% to 54%, and discontinuation for toxicity (approximately 1–13%) and treatment-related deaths were uncommon. SG is associated with predictable, manageable toxicities—principally neutropenia and diarrhea—that infrequently lead to treatment discontinuation when managed with proactive monitoring and dose modification, and with comparatively little severe peripheral neuropathy relative to taxane-based regimens [25,27]. In the palliative setting, where preservation of function and quality of life is paramount, this profile is an important consideration alongside efficacy.

Several limitations temper these conclusions. First and most fundamentally, only one randomized trial provided head-to-head comparative data; the comparative superiority of SG over chemotherapy rests entirely on the ASCENT and could not be pooled across studies. The quantitative synthesis is consequently a single-arm pooling of proportions and reconstructed medians, which is sensitive to between-study clinical heterogeneity and cannot establish causal superiority beyond the trial itself. Second, in the absence of individual patient data, pooled medians were derived from summary statistics, precluding adjustment for line of therapy, prior treatment, and follow-up; OS heterogeneity was high (I^2^ = 75%). Third, the real-world cohorts were retrospective and were rated at moderate-to-serious risk of bias on ROBINS-I, chiefly because of the absence of a comparator and the potential for confounding by indication. Fourth, with fewer than ten cohorts contributing to any outcome, funnel-plot asymmetry and Egger’s test were not formally evaluated, so small-study effects cannot be excluded. Finally, the search was restricted to PubMed/MEDLINE and Web of Science and to reports in English, Spanish, or German; although these databases overlap substantially with Embase and CENTRAL and were supplemented by hand searching and congress screening, the exclusion of Embase, CENTRAL, Scopus, and ClinicalTrials.gov and the language restriction may have left some eligible studies unidentified, and the pooled response and benefit estimates rested on relatively few cohorts. Applying the GRADE framework, we judge the certainty of the evidence to be moderate-to-high for the randomized comparative endpoints and low for the pooled single-arm real-world estimates.

Future work should prioritize individual-patient-data meta-analysis to enable adjusted, time-to-event synthesis; prospective real-world registries with standardized outcome reporting; and trials addressing the questions the current evidence cannot answer—namely the optimal sequencing of Trop-2 ADCs (including SG versus datopotamab deruxtecan and the role of re-treatment after ADC failure), the mechanisms of acquired resistance, and the predictive value of biomarkers beyond Trop-2 expression, whose relationship to benefit remains unclear. Dedicated studies in patients with brain metastases and the integration of SG with first-line immunotherapy also warrant continued investigation.

## 5. Conclusions

This systematic review and meta-analysis confirm that sacituzumab govitecan (SG) is an effective treatment for metastatic triple-negative breast cancer (mTNBC). In the pivotal randomized ASCENT, SG was superior to standard chemotherapy across all efficacy endpoints—progression-free survival (hazard ratio [HR] 0.41, 95% CI 0.33–0.63), overall survival (HR 0.51, 95% CI 0.42–0.64), objective response (odds ratio [OR] 10.3, 95% CI 5.3–19.9), and clinical benefit (OR 7.8, 95% CI 4.7–13.0) [26,27]. Pooling the single-arm experience across clinical trials and six real-world cohorts yielded consistent estimates—an objective response rate of 31%, a clinical benefit rate of 42%, a median progression-free survival of 4.8 months, and a median overall survival of 11.0 months—with low heterogeneity for response and progression-free survival. The close agreement between trial-derived and real-world activity indicates that the activity demonstrated in the controlled setting is broadly consistent in unselected, routinely treated patients, an observation directly relevant to clinical decision-making and health-policy evaluation. Comparative superiority over chemotherapy nonetheless rests on the single ASCENT.

The treatment effect was directionally maintained across historically under-served subgroups, including older patients (progression-free survival HR 0.25) and Black women (HR 0.44); however, the benefit in patients with baseline brain metastases did not reach statistical significance (HR 0.68, 95% CI 0.38–1.23) and should be regarded as hypothesis-generating. Together with a predictable and manageable safety profile dominated by neutropenia and diarrhea, these findings support the established role of SG in second-line and later mTNBC and the broader objective of equitable access to effective therapies. These conclusions should nonetheless be interpreted in light of their principal limitation: comparative evidence derives from a single randomized trial, while the remaining synthesis rests on observational, single-arm data of moderate certainty. As Trop-2-directed antibody–drug conjugates—including SG itself—move into the first-line setting, individual patient data meta-analyses and head-to-head sequencing studies will be needed to define the optimal positioning of these agents within the rapidly evolving mTNBC treatment landscape.

## Figures and Tables

**Table 1:** Characteristics and main efficacy outcomes of the included studies.

Study (Year) [Ref]	Design/Setting	Country	*n* (SG/comp.)	ORR, %	mPFS, mo	mOS, mo
IMMU-132-01 (2019) [25]	Phase I/II, single-arm; ≥3rd line; single-arm (no comparator)	USA	108/-	33.3	5.5	13.0
ASCENT (2021/24) [26,27]	Phase III RCT, 2nd+ line; comparator: TPC (eribulin, vinorelbine, capecitabine, or gemcitabine)	Intl.	267/262	31.1	4.8	11.8
EVER-132-001, Xu (2023) [45] *	Phase IIb, single-arm; ≥3rd line; single-arm (no comparator)	China	80/-	38.8	5.6	NR
Caputo (2024) [46]	Retrospective RW cohort; ≥2nd line; single-arm (no comparator)	Italy	57/-	33.3	4.9	12.4
De Moura (2024) [47]	Retrospective RW cohort; ≥2nd line; single-arm (no comparator)	France	99/-	29.3	3.9	8.6
Alaklabi (2024) [48]	Retrospective RW cohort; ≥2nd line; single-arm (no comparator)	USA	115/-	27.8	4.8	9.6
Hanna (2024) [49]	Retrospective RW cohort; ≥2nd line; single-arm (no comparator)	UK	132/-	NR	5.2	8.7
Püsküllüoğlu (2024) [50]	Retrospective RW cohort; ≥2nd line; single-arm (no comparator)	Poland	79/-	30.4	4.4	10.3
Schäffler (2024) [51]	Retrospective RW cohort; ≥2nd line; single-arm (no comparator)	Germany	43/-	NR	4.9	12.4

SG, sacituzumab govitecan; comp., comparator (treatment of physician’s choice); RCT, randomized controlled trial; RW, real-world; ORR, objective response rate; mPFS/mOS, median progression-free/overall survival; Intl., international; NR, not reported or not reached. * Xu et al. [45] reported OS rates (3/6/9-month survival 93.8%/82.5%/68.0%); median OS was not reached at data cut-off. The line of therapy is indicated for each study in the design/setting column; only ASCENT included a concurrent comparator, namely single-agent treatment of the physician’s choice (eribulin, vinorelbine, capecitabine, or gemcitabine), while all other cohorts were single-arm without a comparator group.

**Table 2:** Reported safety profile of sacituzumab govitecan across the included studies (descriptive summary).

Study (Year) [Ref]	*n* (SG)	Neutropenia G ≥ 3, %	Diarrhea G ≥ 3, %	Febrile Neutropenia, %	Dose Reductions, %	Discontinuation (AE), %	TR Deaths
IMMU-132-01 (2019) [25]	108	43	9	9	NR	3	0
ASCENT, SG arm (2021) [27]	258	51	10	6	22	5	0
EVER-132-001, Xu (2023) [45]	80	62 a	<5 a	7	NR	low	0
Caputo (2024) [46]	57	30 b	NR	NR	39	5	NR
De Moura (2024) [47]	99	hematol. c	5 c	2	17	1	0
Alaklabi (2024) [48]	115	36	7	NR	51	13	NR
Hanna (2024) [49]	132	29	15	NR	54	5	NR
Püsküllüoğlu (2024) [50]	79	43 d	4 d	NR	25	low	NR
Schäffler (2024) [51]	43	33	NR	NR	NR	NR	NR

SG, sacituzumab govitecan; G ≥ 3, grade 3 or higher; AE, adverse event; TR, treatment-related; NR, not reported. Percentages are as reported in each source and are not pooled. a EVER-132-001 (Xu) reported predominantly hematological toxicity: grade ≥3 neutrophil-count decrease ≈ 62%, leukopenia ≈ 45%, and anemia ≈ 22%; grade ≥3 diarrhea was infrequent. b Caputo: grade 3 neutropenia 21.0% plus grade 4 8.7%. c De Moura reported dose-limiting toxicities rather than a full grade ≥3 incidence table; the main limiting toxicity was hematological (mainly neutropenia), with grade 3 diarrhea in five patients and one discontinuation for grade 4 neutropenia. d Püsküllüoğlu reported grade ≥2 (not grade ≥3) adverse events, and treatment discontinuation was predominantly due to disease progression rather than toxicity.

## Data Availability

No new data were created or analyzed in this study.

## Supplementary Materials

The following supporting information can be downloaded at https://www.mdpi.com/article/10.3390/cancers18122005/s1. Supplementary Methods: Extended description of the protocol, registration, search, screening, data-extraction, de-duplication, and statistical procedures; Table S1: Completed PRISMA 2020 checklist; Table S2: Full database-specific search strategies for PubMed/MEDLINE and the Web of Science Core Collection, with line-by-line record counts; Table S3: Risk-of-bias assessment of the ASCENT randomized controlled trial (Cochrane RoB 2); Table S4: Risk-of-bias assessment of the non-randomized studies (ROBINS-I); Table S5: Median progression-free survival (PFS) and overall survival (OS) pooled by study design (clinical trials vs. real-world cohorts), with the test for differences between subgroups; Table S6: GRADE Summary of Findings for the efficacy outcomes of sacituzumab govitecan in metastatic triple-negative breast cancer; Table S7: Mapping of overlapping reports to unique studies (de-duplication; collapsing of the ASCENT and IMMU-132-01 publications into single studies); Table S8: Leave-one-out sensitivity analysis for the pooled single-arm objective response rate.

## Abbreviations

The following abbreviations are used in this manuscript:

ADC	Antibody–drug conjugate
ASCO	American Society of Clinical Oncology
CBR	Clinical benefit rate
CI	Confidence interval
DNA	Deoxyribonucleic acid
DOI	Digital object identifier
ESMO	European Society for Medical Oncology
FDA	US Food and Drug Administration
GRADE	Grading of Recommendations Assessment, Development and Evaluation
HER2	Human epidermal growth factor receptor 2
HR	Hazard ratio
mOS	Median overall survival
mPFS	Median progression-free survival
mTNBC	Metastatic triple-negative breast cancer
NR	Not reported/not reached
OR	Odds ratio
ORR	Objective response rate
OS	Overall survival
OSF	Open Science Framework
PARP	Poly(ADP-ribose) polymerase
PD-L1	Programmed death-ligand 1
PFS	Progression-free survival
PICOS	Population, Intervention, Comparison, Outcomes, Study designs
PRISMA	Preferred Reporting Items for Systematic Reviews and Meta-Analyses
RCT	Randomized controlled trial
RECIST	Response Evaluation Criteria in Solid Tumors (version 1.1)
REML	Restricted maximum likelihood
RoB 2	Risk of bias 2 tool (Cochrane)
ROBINS-I	Risk of bias in non-randomized studies of interventions
RW	Real-world
SABCS	San Antonio Breast Cancer Symposium
SG	Sacituzumab govitecan
SN-38	7-ethyl-10-hydroxycamptothecin (active metabolite of irinotecan)
TNBC	Triple-negative breast cancer
TPC	Treatment of the physician’s choice
Trop-2	Trophoblast cell-surface antigen 2
