# Supplementary material for: Sacituzumab Govitecan in Metastatic Triple-Negative Breast Cancer: A Systematic Review with Meta-Analysis of Single-Arm Efficacy and Integration of Randomized and Real-World Evidence
Source: Cancers (Basel). 2026 Jun 20;18(12):2005. doi: 10.3390/cancers18122005 (PMC13297106; doi:10.3390/cancers18122005)
Supplement: Supplementary file 1 [file cancers-18-02005-s001.zip › cancers-4366623-supplementary.pdf]

Sacituzumab Govitecan in Metastatic Triple-Negative Breast Cancer: A Systematic Review with Meta-Analysis of Single-Arm Efficacy and Integration of Randomized and Real-World Evidence

SUPPLEMENTARY MATERIAL

Reference numbering [n] corresponds to the main manuscript reference list.

Contents

- Supplementary Methods - protocol, registration, and de-duplication
- Table S1. PRISMA 2020 checklist
- Table S2. Database-specific search strategies (PubMed/MEDLINE; Web of Science Core Collection)
- Table S3. Risk-of-bias assessment of the randomized trial (Cochrane RoB 2)
- Table S4. Risk-of-bias assessment of non-randomized studies (ROBINS-I)
- Table S5. Median progression-free survival (PFS) and overall survival (OS) pooled by study design (clinical trials vs real-world cohorts), with a test for differences between subgroups.
- Table S6. Summary of Findings (GRADE)
- Table S7. Mapping of overlapping reports to unique studies (de-duplication)
- Table S8. Leave-one-out sensitivity analysis (pooled single-arm objective response rate)

Supplementary Methods

Protocol and registration. The review was registered in the Open Science Framework (OSF) under DOI 10.17605/OSF.IO/EN5TK. The review followed the PRISMA 2020 statement [28], and the completed checklist is provided in Table S1.

Handling of overlapping reports. Several included trials were described in more than one publication. To preserve statistical independence, all reports of a given trial were collapsed into a single study and counted once; the report used for each estimate is specified in Table S7. The ASCENT trial contributed the comparative (randomized) estimates, taken from the final database-lock report [27]; its prespecified subgroup, biomarker, and secondary analyses [36-42] were used only descriptively. The phase I/II IMMU-132-01 trial was reported in an initial [43] and an expanded [25] analysis and in the regulatory approval summary [44]; the expanded analysis [25] was used for the pooled single-arm estimates.

Table S1. PRISMA 2020 checklist

PRISMA 2020 Checklist

Section and Topic	Item #	Checklist item	Location where item is reported
**TITLE**			
Title	1	Identify the report as a systematic review.	Title
**ABSTRACT**			
Abstract	2	See the PRISMA 2020 for Abstracts checklist.	Abstract
**INTRODUCTION**			
Rationale	3	Describe the rationale for the review in the context of existing knowledge.	Introduction
Objectives	4	Provide an explicit statement of the objective(s) or question(s) the review addresses.	Introduction; Methods (Research question)
**METHODS**			
Eligibility criteria	5	Specify the inclusion and exclusion criteria for the review and how studies were grouped for the syntheses.	Methods (Eligibility criteria)
Information sources	6	Specify all databases, registers, websites, organisations, reference lists and other sources searched or consulted to identify studies. Specify the date when each source was last searched or consulted.	Methods (Information sources); Table S2
Search strategy	7	Present the full search strategies for all databases, registers and websites, including any filters and limits used.	Table S2
Selection process	8	Specify the methods used to decide whether a study met the inclusion criteria of the review, including how many reviewers screened each record and each report retrieved, whether they worked independently, and if applicable, details of automation tools used in the process.	Methods (Study selection)
Data collection process	9	Specify the methods used to collect data from reports, including how many reviewers collected data from each report, whether they worked independently, any processes for obtaining or confirming data from study investigators, and if applicable, details of automation tools used in the process.	Methods (Data extraction)
Data items	10a	List and define all outcomes for which data were sought. Specify whether all results that were compatible with each outcome domain in each study were sought (e.g. for all measures, time points, analyses), and if not, the methods used to decide which results to collect.	Methods (Data extraction; Outcome definitions)
	10b	List and define all other variables for which data were sought (e.g. participant and intervention characteristics, funding sources). Describe any assumptions made about any missing or unclear information.	Methods (Data extraction; Outcome definitions)

Section and Topic	Item #	Checklist item	Location where item is reported
Study risk of bias assessment	11	Specify the methods used to assess risk of bias in the included studies, including details of the tool(s) used, how many reviewers assessed each study and whether they worked independently, and if applicable, details of automation tools used in the process.	Methods (Risk-of-bias assessment); Tables S3-S4
Effect measures	12	Specify for each outcome the effect measure(s) (e.g. risk ratio, mean difference) used in the synthesis or presentation of results.	Methods (Data synthesis)
Synthesis methods	13a	Describe the processes used to decide which studies were eligible for each synthesis (e.g. tabulating the study intervention characteristics and comparing against the planned groups for each synthesis (item #5)).	N.R.
	13b	Describe any methods required to prepare the data for presentation or synthesis, such as handling of missing summary statistics, or data conversions.	N.R.
	13c	Describe any methods used to tabulate or visually display results of individual studies and syntheses.	N.R.
	13d	Describe any methods used to synthesize results and provide a rationale for the choice(s). If meta-analysis was performed, describe the model(s), method(s) to identify the presence and extent of statistical heterogeneity, and software package(s) used.	Methods (Data synthesis)
	13e	Describe any methods used to explore possible causes of heterogeneity among study results (e.g. subgroup analysis, meta-regression).	N.R.
	13f	Describe any sensitivity analyses conducted to assess robustness of the synthesized results.	N.R.
Reporting bias assessment	14	Describe any methods used to assess risk of bias due to missing results in a synthesis (arising from reporting biases).	Methods (Data synthesis); Results
Certainty assessment	15	Describe any methods used to assess certainty (or confidence) in the body of evidence for an outcome.	Methods (Certainty of evidence); Table S6
**RESULTS**			
Study selection	16a	Describe the results of the search and selection process, from the number of records identified in the search to the number of studies included in the review, ideally using a flow diagram.	Results (Study selection); Figure 2
	16b	Cite studies that might appear to meet the inclusion criteria, but which were excluded, and explain why they were excluded.	Results (Study selection); Figure 2
Study characteristics	17	Cite each included study and present its characteristics.	Results; Table 1; Table S7
Risk of bias in studies	18	Present assessments of risk of bias for each included study.	Results; Tables S3-S4
Results of individual studies	19	For all outcomes, present, for each study: (a) summary statistics for each group (where appropriate) and (b) an effect estimate and its precision (e.g. confidence/credible interval), ideally using structured tables or plots.	Results; Table 1; Figures 3-4
Results of syntheses	20a	For each synthesis, briefly summarise the characteristics and risk of bias among contributing studies.	Results; Figures 3-5
	20b	Present results of all statistical syntheses conducted. If meta-analysis was done, present for each the summary estimate and its precision	Results; Figures

Section and Topic	Item #	Checklist item	Location where item is reported
		(e.g. confidence/credible interval) and measures of statistical heterogeneity. If comparing groups, describe the direction of the effect.	3-5
	20c	Present results of all investigations of possible causes of heterogeneity among study results.	Results; Figures 3-5
	20d	Present results of all sensitivity analyses conducted to assess the robustness of the synthesized results.	Results; Figures 3-5
Reporting biases	21	Present assessments of risk of bias due to missing results (arising from reporting biases) for each synthesis assessed.	Results (Heterogeneity, sensitivity, and risk of bias)
Certainty of evidence	22	Present assessments of certainty (or confidence) in the body of evidence for each outcome assessed.	Results / Discussion; Table S6
**DISCUSSION**			
Discussion	23a	Provide a general interpretation of the results in the context of other evidence.	Discussion
	23b	Discuss any limitations of the evidence included in the review.	Discussion
	23c	Discuss any limitations of the review processes used.	Discussion
	23d	Discuss implications of the results for practice, policy, and future research.	Discussion
**OTHER INFORMATION**			
Registration and protocol	24a	Provide registration information for the review, including register name and registration number, or state that the review was not registered.	Methods (Protocol, registration); Supplementary Methods
	24b	Indicate where the review protocol can be accessed, or state that a protocol was not prepared.	Methods (Protocol, registration); Supplementary Methods
	24c	Describe and explain any amendments to information provided at registration or in the protocol.	N.R.
Support	25	Describe sources of financial or non-financial support for the review, and the role of the funders or sponsors in the review.	Funding statement
Competing interests	26	Declare any competing interests of review authors.	Competing interests
Availability of data, code and other materials	27	Report which of the following are publicly available and where they can be found: template data collection forms; data extracted from included studies; data used for all analyses; analytic code; any other materials used in the review.	N.R.

From: Page MJ, McKenzie JE, Bossuyt PM, Boutron I, Hoffmann TC, Mulrow CD, et al. The PRISMA 2020 statement: an updated guideline for reporting systematic reviews. BMJ 2021;372:n71. doi: 10.1136/bmj.n71

Table S2. Database-specific search strategies

PubMed / MEDLINE

#	Query	Records
1	"Triple Negative Breast Neoplasms"[MeSH]	15,273
2	triple-negative breast cancer[tiab] OR triple negative breast neoplasm*[tiab] OR TNBC[tiab]	26,500
3	#1 OR #2	27,881
4	"Immunoconjugates"[MeSH] OR "sacituzumab govitecan"[Supplementary Concept]	16,414
5	sacituzumab govitecan[tiab] OR Trodelvy[tiab] OR IMMU-132[tiab] OR "IMMU 132"[tiab] OR hRS7-SN38[tiab] OR anti-Trop-2 antibody-drug conjugate*[tiab]	756
6	#4 OR #5	16,472
7	#3 AND #6	324
8	#7 AND ("2017/01/01"[PDAT] : "2026/04/30"[PDAT]) AND (English[LA] OR Spanish[LA] OR German[LA]) AND Humans[MH]	269

Web of Science Core Collection

Set	Query	Records
1	TS=("triple negative breast cancer" OR "triple-negative breast neoplasm*" OR TNBC)	47,754
2	TS=("sacituzumab govitecan" OR Trodelvy OR "IMMU-132" OR "IMMU 132" OR hRS7-SN38 OR "anti-Trop-2 antibody-drug conjugate*")	1,930
3	#1 AND #2	667
4	#3 AND PY=(2017-2026) AND LA=(English OR Spanish OR German) AND DT=(Article OR Review OR "Proceedings Paper")	445

Table S3. Risk of bias of the randomized trial (Cochrane RoB 2)

Study	D1: Randomization	D2: Deviations from intended interventions	D3: Missing outcome data	D4: Measurement of the outcome	D5: Selection of reported result	Overall
ASCENT (Bardia 2021/2024) [26,27]	Low	Some concerns	Low	Low	Low	Some concerns

D2 was rated "some concerns" because ASCENT was open-label; however, the co-primary time-to-event outcomes were adjudicated by blinded independent central review, limiting detection bias. The overall judgement ("some concerns") reflects the open-label design only.

Table S4. Risk of bias of non-randomized studies (ROBINS-I)

Study	Confounding	Selection of participants	Classification of interventions	Deviations	Missing data	Measurement of outcomes	Selective reporting	Overall
IMMU-132-01 (Bardia 2019) [25]	Moderate	Moderate	Low	Low	Low	Low	Low	Moderate
EVER-132-001 (Xu 2023) [45]	Moderate	Moderate	Low	Low	Low	Low	Low	Moderate
Caputo 2024 [46]	Serious	Moderate	Low	Moderate	Moderate	Moderate	Low	Serious
De Moura 2024 [47]	Serious	Moderate	Low	Moderate	Moderate	Moderate	Low	Moderate
Alaklabi 2024 [48]	Serious	Moderate	Low	Moderate	Moderate	Moderate	Low	Moderate
Hanna 2024 [49]	Serious	Moderate	Low	Moderate	Moderate	Moderate	Low	Moderate
Püsküllüoğlu 2024 [50]	Serious	Serious	Low	Moderate	Moderate	Moderate	Moderate	Serious
Schäffler 2024 [51]	Serious	Serious	Low	Moderate	Moderate	Moderate	Moderate	Serious

Confounding was the principal limitation: as single-arm and retrospective designs without a concurrent comparator, residual confounding by indication and by prior-treatment burden could not be excluded. Domain judgements summarize the appraisal reported in the main text ("Heterogeneity, sensitivity, and risk of bias") and informed the GRADE ratings in Table S6.

Table S5. Median progression-free survival (PFS) and overall survival (OS) pooled by study design (clinical trials vs real-world cohorts), with a test for differences between subgroups.

Outcome	Subgroup	Studies, n	Pooled median, months (95% CI)	I², %	Test for subgroup differences
PFS	Clinical trials	2	5.1 (4.4-5.8)	—	
PFS	Real-world cohorts	5	4.6 (4.1-5.2)	—	
PFS	Overall	7	4.8 (4.4-5.3)	10	χ^2 = 0.89, df = 1, P = 0.34
OS	Clinical trials	2	12.5 (11.4-13.7)	—	
OS	Real-world cohorts	2	9.1 (7.6-10.9)	—	
OS	Overall	4	11.0 (9.3-13.0)	75	χ^2 = 9.59, df = 1, P = 0.002

Medians were pooled on the natural-logarithmic scale (DerSimonian–Laird random effects) using inverse-variance weights derived from the reported 95% confidence intervals; subtotals correspond to the stratified forest plots in Figure 4. The test for subgroup differences compares the clinical-trial and real-world pooled estimates. PFS was concordant across designs (P = 0.34), whereas OS was significantly lower in the real-world cohorts (P = 0.002), consistent with their more heavily pretreated, less selected populations and supporting a confounding- rather than efficacy-based explanation for the OS difference. Dashes denote subgroup subtotals for which a separate I² is not reported. The overall pooled medians were robust in sensitivity analyses: restricted maximum likelihood gave near-identical estimates (PFS 4.8 months; OS 10.9 months), and leave-one-out analysis produced narrow ranges (PFS 4.7–5.0 months; OS 10.2–11.8 months).

Table S6. Summary of Findings (GRADE)

Patient population: adults with metastatic triple-negative breast cancer. Intervention: sacituzumab govitecan (SG). Part A reports the comparative effect versus treatment of physician's choice (TPC) from the only randomized trial (ASCENT). Part B reports pooled single-arm estimates across trials and real-world cohorts (no concurrent comparator).

A. Comparative outcomes - SG versus chemotherapy (randomized evidence, ASCENT)

Outcome	Participants (studies)	Effect (95% CI)	Certainty	Reasons / comments
Progression-free survival	529 (1 RCT)	HR 0.41 (0.33-0.63)	⊕⊕⊕◯ Moderate	Downgraded once for risk of bias (open-label design); time-to-event outcome supported by blinded independent central review.
Overall survival	529 (1 RCT)	HR 0.51 (0.42-0.64)	⊕⊕⊕⊕ High	Objective outcome not susceptible to open-label assessment; precise estimate.
Objective response rate	529 (1 RCT)	OR 10.3 (5.3-19.9); 31.1% vs 4.2%	⊕⊕⊕◯ Moderate	Downgraded once for risk of bias (open-label, investigator-assessed response despite RECIST 1.1).
Clinical benefit rate	529 (1 RCT)	OR 7.8 (4.7-13.0); 40.4% vs 8.0%	⊕⊕⊕◯ Moderate	Downgraded once for risk of bias (open-label; includes stable disease ≥6 months).

B. Single-arm pooled outcomes - SG activity across trials and real-world cohorts

Outcome	Participants (cohorts)	Pooled estimate (95% CI)	Certainty	Reasons / comments
Objective response rate	≈726 (7 cohorts)	31.1% (28.0-34.4); $I^2=0\%$	⊕⊕◯◯ Low	Non-randomized / single-arm design (starts low); consistent ($I^2=0\%$); few cohorts.
Clinical benefit rate	455 (3 cohorts)	42.2% (37.7-46.8); $I^2=0\%$	⊕⊕◯◯ Low	Non-randomized / single-arm; only three cohorts contributed.
Median progression-free survival	≈766 (7 cohorts)	4.8 mo (4.4-5.3); $I^2=10\%$	⊕⊕◯◯ Low	Pooled summary medians; no individual-patient data; low inconsistency.
Median overall survival	≈480 (4 cohorts)	11.0 mo (9.3-13.0); $I^2=75\%$	⊕◯◯◯ Very low	Further downgraded for serious inconsistency ($I^2=75\%$) reflecting differing prior-treatment burden and follow-up.

GRADE certainty: ⊕⊕⊕⊕ High; ⊕⊕⊕◯ Moderate; ⊕⊕◯◯ Low; ⊕◯◯◯ Very low. RCT-based outcomes start at high certainty; non-randomized/single-arm outcomes start at low certainty. CI, confidence interval; HR, hazard ratio; OR, odds ratio.

Table S7. Mapping of overlapping reports to unique studies

Unique study (identifier)	Design	Reports identified (manuscript ref.)	Use in synthesis
ASCENT (NCT02574455)	Phase III RCT	Bardia 2021 [26]; Bardia 2024 [27]; Hurvitz 2024 [36]; Diéras 2021 [37]; Kalinsky 2021 [38]; Bardia 2021 biomarker [39]; O'Shaughnessy 2022 [40]; Carey 2021 [41]; Carey 2022 [42]	Comparative estimates from final report [27]; subgroups [36-42] descriptive
IMMU-132-01 (NCT01631552)	Phase I/II single-arm	Bardia 2017 [43]; Bardia 2019 [25]; Wahby 2021 FDA summary [44]	Single-arm pooling from expanded analysis [25]
EVER-132-001 (Xu)	Phase IIb single-arm	Xu 2023 [45]	Single-arm pooling
Caputo (Italy)	Retrospective RW cohort	Caputo 2024 [46]	Single-arm pooling
De Moura (France)	Retrospective RW cohort	De Moura 2024 [47]	Single-arm pooling
Alaklabi (USA)	Retrospective RW cohort	Alaklabi 2024 [48]	Single-arm pooling
Hanna (UK)	Retrospective RW cohort	Hanna 2024 [49]	Single-arm pooling
Püsküllüoğlu (Poland)	Retrospective RW cohort	Püsküllüoğlu 2024 [50]	Single-arm pooling
Schäffler (Germany)	Retrospective RW cohort	Schäffler 2024 [51]	Single-arm pooling
NeoSTAR (EXCLUDED)	Neoadjuvant trial	Spring 2024 [35]	Excluded - non-metastatic, early-stage disease

Table S8. Leave-one-out sensitivity analysis (pooled single-arm ORR)

Random-effects (DerSimonian-Laird) pooled objective response rate on the logit scale, recomputed after omitting each contributing cohort. Omission of any single cohort changed the pooled estimate by ≤1.1 percentage points (range 30.2-32.2%), confirming robustness.

Cohort omitted	Pooled ORR (95% CI)
None (all 7 cohorts)	31.1% (28.0-34.4)
ASCENT (SG arm) [27]	31.1% (27.0-35.5)
IMMU-132-01 [25]	30.7% (27.2-34.5)
EVER-132-001 (Xu) [45]	30.2% (26.9-33.6)
Caputo [46]	30.9% (27.4-34.6)
De Moura [47]	31.3% (27.7-35.2)
Alaklabi [48]	32.2% (28.8-35.8)
Püsküllüoğlu [50]	31.2% (27.6-35.0)
